# Impact of HIV testing and treatment services on risky sexual behaviour in the uMgungundlovu District, KwaZulu-Natal, South Africa: a cross-sectional study

**DOI:** 10.1186/s12981-019-0237-z

**Published:** 2019-08-21

**Authors:** Gavin George, Sean Beckett, Cherie Cawood, David Khanyile, Kaymarlin Govender, Ayesha B. M. Kharsany

**Affiliations:** 1 Health Economics and HIV and AIDS Research Division (HEARD), University of KwaZulu-Natal, Westville Campus, J Block, Level 4, University Road, Durban, 4001 South Africa; 2 Centre for the AIDS Programme of Research in South Africa (CAPRISA), Doris Duke Medical Research Institute, Nelson R Mandela School of Medicine, University of KwaZulu-Natal, 2nd Floor, Congella, Private Bag 7, Durban, 4013 South Africa; 3 Epicentre AIDs Risk Management (Pty) Limited, PO Box 3484, Paarl, Cape Town, 7620 South Africa

**Keywords:** HIV prevention, HIV testing services, ART, Risky sexual behaviour, Behaviour change communication

## Abstract

**Introduction:**

The South African public health system plays an important role in the delivery of HIV testing and treatment services. The health system is also an important conduit for targeted behaviour change communication with the expectation that clients who undergo counselling from health personnel, adopt safer sexual practices. Literature remains mixed on the impact these HIV services have on risky sexual behaviour. This analysis examines the sexual behaviour of clients following the utilisation of HIV testing and treatment services in Kwazulu-Natal, South Africa.

**Methods:**

Data were used from two consecutive cross-sectional household surveys undertaken from June 2014 to June 2015 (2014/2015 survey) and from July 2015 to June 2016 (2015/2016 survey) in the uMgungundlovu District of KwaZulu-Natal, South Africa. Collectively, 20,048 randomly selected individuals aged 15 to 49 years old were interviewed across the two surveys. Utilisation of HIV testing and treatment services were used as independent variables and three sexual risk behaviours were used as dependent variables. Multiple regression models assessed the impact HIV testing and treatment services had on sexual risk behaviour while controlling for socio-demographic characteristics.

**Results:**

Having tested for HIV had no association with any of the three sexual risk behaviours. However, receiving an HIV positive diagnosis reduced the likelihood of using condoms inconsistently with the respondents’ most recent partner (AOR: 0.64; 95% CI 0.54–0.77). Antiretroviral use was negatively associated with inconsistent condom use (AOR: 0.45; 95% CI 0.35–0.58) and number of sexual partners in the previous year (AOR: 0.61; 95% CI 0.46–0.81).

**Conclusions:**

Results indicate that HIV testing and treatment services and the assumed exposure of clients to behaviour change communication, had a limited effect in reducing risky sexual behaviour. Data suggests that the engagement between health personnel and individuals accessing HIV testing and treatment services does not necessarily translate into the adoption of safer sexual practices, with the exception of individuals testing positive for HIV and those on ARV treatment, who had adopted safer sexual practices.

**Electronic supplementary material:**

The online version of this article (10.1186/s12981-019-0237-z) contains supplementary material, which is available to authorized users.

## Background

South Africa has the largest national HIV treatment programme in the world with 3.7 million people having tested and initiated on antiretroviral treatment as at the end of 2016 (ART) [1]. Testing and treatment uptake rates continue to increase with the adoption of universal test and treat (UTT) aimed at initiating patients early on lifelong ART treatment in an effort to minimise onward HIV transmission [1]. The Fifth South African National HIV Prevalence, Incidence, Behaviour and Communication Survey (SABSSM) conducted in 2017 estimated that 84.9% of the adult population living with HIV (15 to 64 years) know their status, whilst 70.6% had initiated on ART [2].

Whilst biomedical prevention interventions, together with the increased number of people on treatment, have led to a reduction of HIV incidence [3–6], risky sexual behaviour appears to be on the increase in South Africa with successive nation-wide surveys revealing high levels of inconsistent condom use, evidence of multiple and concurrent partners, and the increased prevalence of age-disparate partnerships [2, 7]. This, despite survey respondents indicating that HIV services, including HIV testing services (HTS), were easily accessible and further evidenced by the majority who had previously tested for HIV [2]. All patients accessing public health services should be encouraged to test for HIV as per national guidelines, with pre and post-test counselling forming part of HTS as guided by the South African national HIV counselling and testing policy [8]. Counselling is undertaken by trained professional health workers. Health workers performing the counselling function would have undertaken counsellor training in accordance with National Minimum Standards for HIV Counselling and Testing (HCT) [8]. Persons completing approved training on HCT receive competency certificates, with refresher training encouraged after 36 months (assuming continuous provision of HCT) or recertification for persons who have not conducted HCT for more than 12 months [8]. Counselling is expected to promote behavioural change, educating clients on ways to prevent HIV transmission and promoting treatment adherence for person’s initiated onto ART [9, 10]. Literature on the effectiveness of testing and HIV treatment services with regards to reducing risky sexual behaviour has however drawn varied conclusions [11–19].

Studies have found only a moderate positive influence on risky sexual behaviour following access to HTS [12], with others indicating no impact on risky sexual behaviour following utilisation of HTS [11]. There is evidence that clinician-delivered counselling is effective in reducing unprotected sex among people testing positive for HIV [13], whilst further studies have revealed no impact for individuals testing HIV negative [18]. A further study has revealed that exposure to HTS (compared to non-exposure) was associated with a 41% reduction in the hazard of HIV acquisition among youth in KwaZulu-Natal over 4.5 years [19]. Systematic reviews have also highlighted the mixed evidence on the effect ART initiation has had on reducing risky sexual behaviour in developing countries, whilst indicating that few rigorous studies on their linkages exist [17]. Research assessing risky sexual behaviour amongst individuals on ART in the South African context also remain mixed, with studies indicating either no effect on risky sexual behaviour [20], decreased risk behaviour for those on ART compared to those not on treatment [21–23] or an increase in risky sexual behaviour amongst individuals on ART [24–26]. The research on risky sexual behaviours reviewed above has focused on individuals already enrolled on ART with little evidence on ART-naive persons (HIV-infected individuals not on ART) [27]. The comparison therefore, between HIV-positive individuals on ART and ART-naive remains largely under researched. A better understanding of the underlying mechanisms and correlations of high-risk sexual behaviour among persons utilising HTS and those on and yet to initiate on ART remains a priority for researchers and public health professionals.

This study contributes to the current literature drawing from data collected from two cross-sectional community level surveys examining sexual behaviour of individuals who have accessed HIV testing and treatment services. The aim is to assess whether respondents who, having accessed testing and treatment services, and thus presumably been exposed to Behaviour Change Communication (BCC) through health personnel administered counselling, exhibit less risky sexual behaviour. The study posits that reduced risky sexual behaviour could be attributed to the exposure to BCC encountered through the engagement with HIV services, specifically HTS, and following ART initiation.

## Methods

### Setting and study design

This study used data from the HIV Incidence Provincial Surveillance System (HIPSS) [28]. The surveillance area is located in Vulindlela and the neighboring greater Edendale area located in the uMgungundlovu District of KwaZulu-Natal, South Africa. The study surveillance area consisted of an estimated 95,641 households with an estimated 367,906 inhabitants. The settlement types located in the surveillance area may be described as rural (Vulindlela) and peri-urban (Edendale). The area is characterised by high levels of poverty, poor infrastructure and high levels of HIV prevalence. Two consecutive cross-sectional household surveys were undertaken from June 2014 to June 2015 (2014/2015 survey) and from July 2015 to June 2016 (2015/2016 survey). The surveys were administered by fieldworkers to the study participants.

### Study participants, sampling procedure and sample size

Households were randomly selected using a two-stage cluster-based random sampling method. Enumeration areas (EA) were identified and sampled initially, with households systematically drawn from each EA. One individual aged 15 to 49 years, was randomly selected and enrolled into the study [28]. The 2014/2015 survey had a sample of 9812 (86.9% response rate) individuals selected from 11,289 households whilst the 2015/2016 survey had 10,236 individuals (83.6% response rate) selected from 12,247 households.

### Measures

Having tested for HIV, and if HIV positive, having initiated treatment, were selected as independent variables to assess the impact of HIV testing and treatment services on risky sexual behaviour. Additionally, we analysed the impact of the test result following HIV testing on an individual’s sexual behaviour. The independent variables selected were whether a respondent had ever tested for HIV (0 = Never tested for HIV, 1 = Tested for HIV), HIV status of the individual following their HIV test (0 = HIV negative, 1 = HIV positive,), and self-reported ART uptake (0 = Not on ART, 1 = On ART).

Additional analyses investigated the impact multiple HIV tests may have had on the individuals risky sexual behaviour, measured by asking the individual how many HIV tests they have had (1 = 1 HIV test, 2 = 2 HIV tests, 3 = 3 HIV tests, 4 = 4 HIV tests and 5 = five or more HIV tests); and the impact of three different types of support granted to HIV positive individuals on their sexual risk behaviours, namely; emotional support (0 = no emotional support, 1 = received emotional support), support from a treatment buddy (0 = didn’t have an ARV treatment buddy, 1 = had an ARV treatment buddy) and lastly, home-based support (0 = Didn’t receive home-based support, 1 = received home-based support). These three support types dominated those listed by those individuals on ART. In all there were five separate independent variables in the analysis.

There were three dependent variables used to operationalise sexual risk behaviour: condom use with most recent sexual partner in the last 12 months (0 = consistent condom use/always used condoms; 1 = inconsistent condom use/sometimes or never used condoms); number of sexual partners in the previous year (0 = 0 to 1 sexual partner in the previous year; 1 = 2 or more sexual partners in the previous year); engaging in transactional sex (0 = not giving or receiving goods or money for sex in the previous year; 1 = have given or received goods or money for sex in the previous year). These variables have been used previously as markers for risky sexual behaviour [29–34].

The control variables used in the multivariate analysis were age (coded as: 1 = 15 to 19 years, 2 = 20 to 24 years, 3 = 25 to 34 years and 4 = 35 to 49 years), sex (coded as: 1 = male and 2 = female), marital status (coded as: 0 = unmarried and 1 = married), highest level of formal education attained (coded as: 1 = incomplete secondary schooling, 2 = completed secondary schooling and 3 = some tertiary education), household food security (coded as: 1 = food secure, 2 = moderate food insecurity, 3 = severe food insecurity) [35], being away from home for more than 1 month consecutively in the previous 12 months (coded as: 1 = yes, 2 = no), knowledge of HIV status of most recent sexual partner (coded as: 1 = partner is HIV positive, 2 = partner is HIV negative, 3 = don’t know partner’s HIV status) and duration of relationship of most recent partner (continuous variable coded as number of years). The latter two control variables were only used in the condom use regression models.

### Data analysis

The two cross-sectional databases were analysed separately and all results are presented separately. Weighted descriptive statistics are provided on the distribution of the sample by socio-demographic variables and HIV services accessed (see Table 1 for full list of variables). Additionally, weighted descriptive statistics are provided on the sexual risk behaviours. Thirty multiple logistic regression models (15 for each database) were constructed to assess the potential impact of the selected HIV interventions on risky sexual behaviour. All models controlled for confounding factors. To correct for unequal selection probabilities and to compensate for non-response, sample weights were applied. Each database was weighted separately and the standard errors were adjusted for the sampling design. All analyses were conducted in SPSS version 25.

**Table 1:** Weighted percentages and unweighted sample sizes for demographic, SES and HIV service utilisation in Edendale and Vulindlela, KwaZulu-Natal, South Africa for 2014/2015 survey (n = 9812) and 2015/2016 survey (n = 10,236)

	Survey 2014/2015		Survey 2015/2016	
	%	n	%	n
Sex				
Male	48.2	3547	48.2	3895
Female	51.8	6265	51.8	6341
Age of respondent				
15–24 years	39.0	3696	39.0	4107
25–34 years	34.4	3197	34.4	3549
35–49 years	26.6	2919	26.6	2580
Highest education level attained				
Didn’t finish sec. school^b^	54.9	5246	54.8	5577
Completed sec. school	39.8	4009	38.9	3979
Tertiary education	5.3	552	6.3	677
Food security in household				
Food insecure	18.2	1740	39.4	3998
Moderate food insecurity	9.1	880	19.3	1996
Food secure	72.7	7192	41.3	4242
Main income in the household^a^				
Salary/wage	52.7	5194	58.2	5974
Pension/grant	28.1	2625	32.1	3258
No income	7.3	762	3.1	314
Current marital status				
Married	8.9	862	10.9	1106
Unmarried	91.1	8950	89.1	9130
> 1 mo. away from home in previous 12 mo.				
Yes	10.4	1021	8.0	815
No	89.6	8755	92.0	9412
Ever tested for HIV				
Did not test	24.5	2547	13.1	1184
Tested for HIV	75.5	7265	86.9	9047
Number of HIV tests in previous 12 mo.				
1	28.3	2071	24.1	2075
2	20.9	1477	18.7	1666
3	19.4	1398	18.0	1661
4	9.8	770	10.7	981
≥ 5	21.6	1483	28.5	2664
Self-reported HIV status				
Negative	70.0	4708	71.1	6156
Positive	30.0	2367	28.9	2763
HIV status (laboratory derived)				
Negative	63.7	5843	64.8	6366
Positive	36.3	3969	35.2	3870
ART take-up				
Not on ART	57.7	2377	44.7	1695
On ART	42.3	1592	55.3	2175
Received emotional support				
No	61.0	1459	53.4%	1498
Yes	39.0	908	46.6%	1265
Had a treatment buddy				
No	78.0	1822	71.3%	1978
Yes	22.0	545	28.7%	785
Received home-based care				
No	89.6	2138	78.0%	2160
Yes	10.4	229	22.0%	603

^a^Only three most prevalent forms of household income reported for the sake of brevity

^b^This includes individuals who have no schooling, only primary schooling or incomplete secondary schooling. These categories were collapsed as the level of vulnerability is similar for all individuals who don’t complete secondary schooling

## Results

Table 1 details the distribution of the sample by sociodemographic characteristics and HIV service utilisation. There were slightly more females (51.8%) in both surveys, with almost a third (31.9%) of respondents aged between 25 and 34 years old and 26.6% aged between 35 and 49 years. The two main variables used for weighting these data were age and sex, hence the demographics are the same for both surveys. The majority (54.9% in the 2014/2015 survey and 54.8% in 2015/2016 survey) of the sample had not completed schooling and only a minority of individuals had some tertiary education (5.3% in the 2014/2015 survey and 6.3% 2015/2016 survey). However, the sample included 15 to 17-year-old individuals who were not expected to have completed secondary or entered tertiary schooling. The vast majority (91.1% in the 2014/2015 survey and 89.1% in the 2015/2016 survey) of respondents were unmarried. Food insecurity more than doubled between the 2014/2015 and 2015/2016 survey (18.2% and 39.4% respectively). Just more than half of the households indicated that a salary or wage was the primary source of income (52.7% in 2014/2015 survey and 58.2% in the 2015/2016 survey). A minority of individuals in the sample had spent more than 1 month away from home in the previous year (10.4% in 2014/2015 survey and 8.0% in the 2015/2016 survey).

The vast majority of study participants had tested previously for HIV (75.5% in 2014/2015 survey and 86.9% in the 2015/2016 survey). Of those who had tested, more than a quarter (28.3%) in the 2014/2015 survey had tested once, with this decreasing to less than a quarter (24.1%) having only tested once in the 2015/2016 survey. More than one third of the study respondents were living with HIV (36.3% in 2014/2015 survey and 35.2% in 2015/2016 survey) as diagnosed from their peripheral blood samples. There was a large increase in the number of HIV positive (laboratory-diagnosed) individuals on ART (self-reported) from the 2014/2015 survey to the 2015/2016 survey (42.3% vs. 55.3% respectively). The most prevalent form of support for HIV positive people was emotional support (39.0% in 2014/2015 survey and 46.6% in 2015/2016 survey).

Table 2 highlights the risky sexual practices of study participants according to study survey. The vast majority of respondents used condoms inconsistently with their most recent partner (78.4% in the 2014/2015 survey and 81.2% in the 2015/2016 survey). The median number of sexual partners in the previous 12 months was one partner in both surveys (2014/2015 survey range: 0–26 partners; 2015/2016 survey range: 1–50 partners). A minority of respondents had two or more partners and used condoms inconsistently with the most recent partner (11.6% in the 2014/2015 survey and 15.5% in the 2015/2016 survey). Few respondents had engaged in transactional sex with their most recent sexual partner (13.9% in the 2014/2015 survey and 11.1% in 2015/2016 survey).

**Table 2:** Weighted percentages and unweighted sample sizes for sexual risk behaviours for the study sample in Edendale and Vulindlela, KwaZulu-Natal, South Africa for the 2014/2015 survey (n = 9812) and 2015/2016 survey (n = 10,236)

	Survey 2014/2015		Survey 2015/2016	
	%/median	N	%/median	N
% inconsistent condom use with most recent partner	78.4	6619	81.2	7301
Median (IQR) number sex partners in previous 12 mo.	1 (1)	6145	1 (1)	7830
% exchanged money/gifts for sex with previous partner	13.9	1383	11.1	1041

Table 3 presents the multiple regression results for HIV testing and the three risk behaviours disaggregated by each survey, controlling for a number of socio-demographic variables. All odds ratios have been adjusted for age, sex, marital status, educational attainment, being away from home for more than 1 month consecutively in the previous 12 months and food security. The condom use models include knowledge of partner’s HIV status and duration of relationship. The results indicate that respondents in the 2014/2015 survey who had ever tested for HIV were more likely to use a condom consistently (Model 1, AOR: 0.62, 95% CI 0.49–0.78, p < 0.001) than those who had never tested. Those individuals who tested for HIV were also less likely to engage in transactional sex (Model 2, AOR: 0.62, 95% CI 0.50–0.77, p < 0.001) than those in the 2014/2015 survey who had never tested. However, in the 2015/2016 survey, respondents who had tested showed no significant differences in risky sexual behaviour from respondents who hadn’t tested for HIV in the previous 12 months.

**Table 3:** Multiple logistic regression models for the association between HIV testing and four sexual risk behaviours (adjusted odds ratios with 95% confidence intervals in parentheses) in Edendale and Vulindlela, KwaZulu-Natal, survey period 2014 to 2016

	Survey 2014/2015			Survey 2015/2016		
	[1]^a^ Condom use last partner^b^	[2] Transaction sex^c^	[3] Sex partners 12 mo.^d^	[4] Condom use last partner^b^	[5] Transaction sex^c^	[6] Sexual partners 12 mo. ^d^
HIV tested (vs. did not test)	0.62*** (0.49–0.78)	0.62*** (0.50–0.77)	1.18 (0.89–1.55)	0.91 (0.72–1.14)	1.07 (0.78–1.48)	0.81 (0.65–1.02)
15–24 years (vs. 35–49 years)	0.66*** (0.54–0.81)	0.84 (0.68–1.04)	2.14*** (1.57–2.92)	1.00 (0.83–1.20)	0.98 (0.78–1.22)	1.79*** (1.43–2.24)
25–34 years (vs. 35–49 years)	1.06 (0.87–1.29)	1.12 (0.93–1.35)	1.90*** (1.40–2.57)	1.32** (1.10–1.58)	1.16 (0.95–1.42)	1.62*** (1.30–2.01)
Male (vs. female)	0.75*** (0.64–0.88)	0.79* (0.65–0.96)	7.01*** (5.53–8.88)	0.76*** (0.67–0.87)	0.96 (0.83–1.12)	4.62*** (3.93–5.44)
Married (vs. unmarried)	1.24*** (0.90–1.72)	0.66** (0.50–0.88)	0.23*** (0.13–0.41)	1.09 (0.86–1.39)	0.63*** (0.48–0.84)	0.36 (0.84–1.51)
Incomplete sec. school (vs. tertiary)	1.51* (1.07–2.14)	2.13*** (1.40–3.23)	0.70 (0.43–1.14)	1.29* (1.01–1.65)	1.05 (0.74–1.48)	1.12 (0.84–1.51)
Complete sec. school (vs. tertiary)	1.33 (0.96–1.85)	1.46 (0.96–2.20)	0.58* (0.38–0.90)	1.10 (0.87–1.39)	1.10 (0.80–1.51)	1.13 (0.85–1.49)
away > 1 mo. in previous year (vs. < 1mo. previous year)	1.21 (0.89–1.63)	1.29 (0.98–1.72)	0.94 (0.64–1.38)	0.92 (0.71–1.18)	0.93 (0.67–1.30)	1.62*** (1.27–2.07)
Highly food insecure (vs. food secure)	0.85 (0.68–1.06)	0.52*** (0.38–0.70)	1.31 (0.99–1.74)	0.87 (0.75–1.02)	0.50*** (0.36–0.70)	1.31** (1.08–1.61)
Moderate food insecure (vs. food secure)	1.04 (0.78–1.39)	0.75 (0.52–1.10)	1.26 (0.82–1.93)	0.78** (0.65–0.93)	0.48*** (0.33–0.70)	1.41*** (1.12–1.78)
Partner HIV positive (vs. don’t know partner status)	0.59*** (0.42–0.84)	–	–	1.06 (0.77–1.45)	–	–
Partner not HIV positive (vs. don’t know partner status)	1.11 (0.88–1.39)	–	–	1.23 (0.94–1.60)	–	–
Duration of last relationship (continuous)	1.03*** (1.01–1.05)	–	–	1.09*** (1.07–1.10)	–	–
Pseudo R^2^	0.05	0.04	0.20	0.07	0.03	0.17
n	7967	8271	6121	8860	8887	7819

^a^The numbers in the brackets refer to the multivariate regression model number ***p < 0.001, **p < 0.01, *p < 0.05

^b^Condom use with most recent sexual partner in the last 12 months (0 = consistent condom use/always used condoms; 1 = inconsistent condom use/sometimes or never used condoms)

^c^Engaging in transactional sex (0 = not giving or receiving goods or money for sex in the previous year; 1 = have given or received goods or money for sex in the previous year)

^d^Number of sexual partners in the previous year (0 = 0 to 1 sexual partner in the previous year; 1 = 2 or more sexual partners in the previous year)

To assess if undergoing more than one HIV test (dosage effect) had an effect on the sexual risk behaviours of individuals (see Additional file 1: Table S1) we regressed the number of HIV tests against the three sexual risk variables. The results indicate that for the 2014/2015 survey, there was no consistent effect on inconsistent condom use with their last partner; however, individuals who had undergone only one HIV test were more likely to engage in transactional sex (Model 20, AOR: 1.71, 95% CI 1.27–2.30, p < 0.001) than individuals who had five or more tests. In contrast to the previous finding, individuals who had one HIV test had fewer sexual partners in the previous 12 months (Model 21, AOR: 0.66, 95% CI 0.46–0.95, p < 0.05) than individuals who had tested five or more times. The 2015/2016 survey shows that those who underwent two (Model 22, AOR: 1.36, 95% CI 1.11–1.66, p < 0.01) or four HIV tests (Model 22, AOR: 1.26, 95% CI 1.01–1.58, p < 0.05) were more likely to use condoms inconsistently than those who underwent five or more HIV tests. The same results were found for transactional sex, with individuals who tested twice (Model 23, AOR: 1.75, 95% CI 1.27–2.40, p < 0.001) or thrice (Model 23, AOR: 1.76, 95% CI 1.33–2.33, p < 0.001) more likely to engage in transactional sex than those who underwent five or more HIV tests. The rest of the results for the 2015/2016 survey were mixed and did not present a coherent picture on the effect multiple HIV tests had on individual’s sexual risk behaviours.

Table 4 presents the multiple regression results for respondents who knew their HIV status and the risk behaviours separated by the 2014/2015 and 2015/2016 survey. The results revealed that inconsistent condom use is negatively associated with HIV status in the 2014/2015 survey (Model 7, AOR: 0.46, 95% CI 0.37–0.57, p < 0.001). This relationship was replicated in 2015/2016 survey (Model 10, AOR: 0.64, 95% CI 0.54–0.77, p < 0.001).

**Table 4:** Multiple logistic regression models for the association between HIV status knowledge and three sexual risk behaviours (adjusted odds ratios with 95% confidence intervals in parentheses) in Edendale and Vulindlela, KwaZulu-Natal, survey period 2014 to 2016

	Survey 2014/2015			Survey 2015/2016		
	[7]^a^ Condom use last partner^b^	[8] Transaction sex^c^	[9] Sex partners 12 mo.^d^	[10] Condom use last partner^b^	[11] Transaction sex^c^	[12] Sexual partners 12 mo.^d^
HIV positive (vs. HIV negative)	0.46*** (0.37–0.57)	1.09 (0.85–1.39)	0.90 (0.63–1.29)	0.64*** (0.54–0.77)	1.09 (0.90–1.31)	0.87 (0.69–1.09)
15–24 years (vs. 35–49 years)	0.57*** (0.45–0.73)	0.85 (0.64–1.12)	1.86*** (1.24–2.78)	0.90 (0.74–1.11)	1.05 (0.83–1.32)	1.7*** (1.32–2.18)
25–34 years (vs. 35–49 years)	1.03 (0.83–1.27)	1.08 (0.86–1.35)	1.86*** (1.31–2.65)	1.28* (1.07–1.54)	1.2 (0.96–1.49)	1.54*** (1.23–1.93)
Male (vs. female)	0.72** (0.60–0.87)	0.9 (0.72–1.13)	6.36*** (4.87–8.30)	0.70*** (0.61–0.80)	0.98 (0.84–1.15)	4.35*** (3.67–5.16)
Married (vs. unmarried)	1.17 (0.83–1.65)	0.63* (0.45–0.88)	0.18*** (0.09–0.36)	1.09 (0.85–1.38)	0.67* (0.50–0.90)	0.31*** (0.21–0.46)
Incomplete sec. school (vs. tertiary)	1.56* (1.11–2.20)	2.36*** (1.40–3.98)	0.65 (0.39–1.07)	1.33* (1.04–1.71)	1.01 (0.72–1.41)	1.21 (0.90–1.65)
Complete sec. school (vs. tertiary)	1.31 (0.95–1.81)	1.78* (1.08–2.92)	0.52*** (0.34–0.81)	1.14 (0.89–1.45)	1.11 (0.82–1.51)	1.17 (0.87–1.57)
away > 1 mo. in previous year (vs. < 1mo. previous year)	1.24 (0.90–1.70)	1.24 (0.89–1.73)	0.94 (0.61–1.43)	0.88 (0.68–1.14)	0.83 (0.59–1.17)	1.52*** (1.17–1.96)
Highly food insecure (vs. food secure)	0.87 (0.68–1.10)	0.55*** (0.40–0.76)	1.17 (0.87–1.59)	0.85 (0.73–1.00)	0.52*** (0.37–0.74)	1.28* (1.03–1.59)
Moderate food insecure (vs. food secure)	1.04 (0.77–1.40)	0.76 (0.52–1.13)	1.31 (0.86–2.01)	0.73** (0.61–0.88)	0.46*** (0.31–0.67)	1.42* (1.11–1.81)
Partner HIV positive (vs. don’t know partner status)	0.74 (0.51–1.09)			1.21 (0.88–1.66)		
Partner not HIV positive (vs. don’t know partner status)	0.92 (0.69–1.23)			1.12 (0.85–1.48)		
Duration of last relationship (continuous)	1.03* (1.01–1.05)			1.08*** (1.07–1.10)		
Pseudo R^2^	0.06	0.03	0.20	0.07	0.03	0.16
n	6205	6436	5074	8035	8059	7113

^a^The numbers in the brackets refer to the Multivariate regression model number. ***p < 0.001, **p < 0.01, *p < 0.05

^b^Condom use with most recent sexual partner in the last 12 months (0 = consistent condom use/always used condoms; 1 = inconsistent condom use/sometimes or never used condoms)

^c^Engaging in transactional sex (0 = not giving or receiving goods or money for sex in the previous year; 1 = have given or received goods or money for sex in the previous year)

^d^Number of sexual partners in the previous year (0 = 0 to 1 sexual partner in the previous year; 1 = 2 or more sexual partners in the previous year)

Table 5 presents the multiple regression results for individuals on ART and the three sexual risk behaviours in both the 2014/2015 and 2015/2016 surveys. The results reveal that there was a negative association between ART use and inconsistent condom use in the previous 12 months in the 2014/2015 survey (Model 13, AOR: 0.45, 95% CI 0.35–0.58, p < 0.001). The same result was found in 2015/2016 survey (Model 16, AOR: 0.54, 95% CI 0.44–0.67, p < 0.001). The results also indicate that there was a negative association between ART use and number of sexual partners in the previous 12 months only in the 2015/2016 survey (Model 18, AOR: 0.63, 95% CI 0.48–0.83, p < 0.001).

**Table 5:** Multiple logistic regression models for the association between accessing HIV treatment and three sexual risk behaviours (adjusted odds ratios with 95% confidence intervals in parentheses) in Edendale and Vulindlela, KwaZulu-Natal, survey period 2014 to 2016

	Survey 2014/2015			Survey 2015/2016		
	[13]^a^ Condom use last partner^b^	[14] Transaction sex^c^	[15] Sex partners 12 mo.^d^	[16] Condom use last partner^b^	[17] Transaction sex^c^	[18] Sexual partners 12 mo.^d^
On ART (vs. not on ART)	0.45*** (0.35–0.58)	0.83 (0.63–1.08)	0.87 (0.57–1.33)	0.54*** (0.44–0.67)	0.95 (0.73–1.22)	0.63*** (0.48–0.83)
15–24 years (vs. 35–49 years)	0.66* (0.48–0.91)	0.88 (0.64–1.22)	2.09* (1.11–3.95)	1.29 (0.92–1.79)	1.12 (0.77–1.62)	1.45 (0.98–2.16)
25–34 years (vs. 35–49 years)	1.09 (0.86–1.40)	0.91 (0.70–1.17)	1.89* (1.19–2.99)	1.46*** (1.18–1.82)	1.32* (1.04–1.69)	1.21 (0.9–1.62)
Male (vs. female)	0.91 (0.70–1.18)	0.95 (0.75–1.2)	6.16*** (4.43–8.58)	0.79* (0.63–0.98)	1.03 (0.80–1.32)	4.16*** (3.07–5.65)
Married (vs. unmarried)	0.67 (0.44–1.01)	0.58* (0.37–0.89)	0.18*** (0.07–0.46)	0.84 (0.62–1.14)	0.71 (0.48–1.03)	0.35*** (0.20–0.64)
Incomplete sec. school (vs. tertiary)	1.52 (0.82–2.81)	1.95* (1.10–3.47)	0.78 (0.34–1.78)	1.36 (0.86–2.15)	0.66 (0.38–1.12)	1.11 (0.63–1.97)
Complete sec. school (vs. tertiary)	1.25 (0.68–2.29)	1.37 (0.79–2.37)	0.68 (0.29–1.61)	1.03 (0.63–1.67)	0.71 (0.42–1.19)	0.94 (0.52–1.71)
away > 1 mo. in previous year (vs. < 1mo. previous year)	1.59* (1.08–2.33)	1.45 (1.00–2.09)	0.97 (0.58–1.62)	0.79 (0.53–1.17)	0.98 (0.6–1.6)	1.79* (1.18–2.71)
Highly food insecure (vs. food secure)	0.85 (0.64–1.11)	0.59* (0.41–0.86)	1.39 (0.89–2.17)	0.78* (0.62–0.98)	0.56*** (0.38–0.83)	1.32 (0.96–1.82)
Moderate food insecure (vs. food secure)	0.83 (0.55–1.26)	1.00 (0.64–1.56)	1.07 (0.54–2.1)	0.71* (0.54–0.94)	0.61* (0.38–0.95)	1.29 (0.86–1.93)
Partner HIV positive (vs. don’t know partner status)	0.74 (0.49–1.13)	–	–	1.09 (0.74–1.62)	–	–
Partner not HIV positive (vs. don’t know partner status)	0.89 (0.65–1.22)	–	–	0.89 (0.62–1.28)	–	–
Duration of last relationship (continuous)	1.01 (0.99–1.03)	–	–	1.07*** (1.05–1.10)	–	–
Pseudo R^2^	0.06	0.03	0.19	0.07	0.03	0.15
n	3634	3756	2737	3763	3776	3268

^a^The numbers in the brackets refer to the Multivariate regression model number. ***p < 0.001, **p < 0.01, *p < 0.05

^b^Condom use with most recent sexual partner in the last 12 months (0 = consistent condom use/always used condoms; 1 = inconsistent condom use/sometimes or never used condoms)

^c^Engaging in transactional sex (0 = not giving or receiving goods or money for sex in the previous year; 1 = have given or received goods or money for sex in the previous year)

^d^Number of sexual partners in the previous year (0 = 0 to 1 sexual partner in the previous year; 1 = 2 or more sexual partners in the previous year)

We further assessed how three forms of treatment support (emotional support, an ART treatment buddy and home-based support) impacted sexual risk behaviours of ART patients. The results for these multiple regressions are presented in Additional file 1: Table S2. The results indicate that in the 2014/2015 survey, those individuals who had a treatment buddy were less likely to engage in transactional sex (Model 26, AOR: 0.60, 95% CI 0.38–0.96, p < 0.05). In the 2015/2016 survey the same result for treatment buddy and transactional sex (Model 29, AOR: 0.31, 95% CI 0.14–0.65, p < 0.001) was found. In addition, those who had received emotional support were less likely to engage in transactional sex than those who had not received emotional support (Model 29, AOR: 0.38, 95% CI 0.23–0.65, p < 0.001). HIV positive individuals who received emotional support (Model 30, AOR: 0.65, 95% CI 0.43–0.97, p < 0.05) or had a treatment buddy (Model 30, AOR: 0.46, 95% CI 0.26–0.79, p < 0.05) were also less likely to have two or more sexual partners in the previous 12 months than study respondents who had not received emotional support or didn’t have a treatment buddy in the 2015/2016 survey. Contrastingly, HIV positive individuals in the 2015/2016 survey who had received home-based care appeared more likely to use condoms inconsistently (Model 29, AOR: 2.24, 95% CI 1.56–3.23, p < 0.001), to engage in transactional sex (Model 30, AOR: 5.40, 95% CI 3.11–9.39, p < 0.001), and more likely to have two or more sexual partners in the previous year (Model 30, AOR: 1.97, 95% CI 1.16–3.36, p < 0.05) than those who had not received home-based care.

## Discussion

The findings of this study reveals mixed evidence on the effectiveness testing and treatment services have had on reducing risky sexual behaviour. These results affirm previous evidence, which has presented mixed and often negative results regarding the impact exposure to health-system-based interventions have had on reducing risky sexual behaviour [11–19]. Data from this study reveals that the biggest influence in reducing risky sexual behaviour is whether an individual is aware of their HIV positive status and whether they initiated onto HIV treatment, which previous South African studies have also found [21–23]. Specifically, the data revealed an increased likelihood of consistent condom use with the most recent sexual partner after having received a positive result following an HIV test in the 2014/2015 survey. Similarly, respondents on ART (compared to ART-naive respondents) were more likely to use condoms consistently with their partner and were less likely to have had two or more partners in the previous 12 months. These results echo those found in a Ugandan study, indicating that ART-naive individuals were more likely to exhibit risky sexual behaviours than individuals on treatment [27].

However, these positive effects were not sustained in the 2015/2016 survey. Notably, the 2015/2016 survey revealed greater HIV service utilisation, with more respondents having both tested and initiated onto treatment. Whilst this increased utilisation is encouraging, the diminished influence of these services in reducing risky sexual behaviour is of concern. This study also revealed mixed results about the effect treatment support has had on risky sexual behaviour. Individuals on treatment who had access to treatment buddies and emotional support indicated lower levels of risky sexual behaviour in both surveys. Contrastingly, individuals receiving home-based care indicated increased levels of risky sexual behaviour. These results are difficult to interpret given we are unaware of the form and content of these treatment support interventions and the screening process determining which ART patients enrolled into which of these interventions. High risk ART patients may have been targeted for enrolment into support interventions, whilst home-based care may have simply comprised of medication drop-offs for stable patients, thus minimising opportunities to meaningfully engage with respondents and therefore effect behaviour change. Home-based patients may also have been attended by lower level health cadres such as community health workers (CHWs) or lay counsellors. Whilst the National HCT guidelines (2015) indicate that all health cadres performing counselling services are to be trained, the subsequent delivery of counselling services may yet yield different effects depending on the different health worker cadres. Further research is therefore required on the effect counselling offered by different cadres of health workers have on patient’s risky sexual behaviour. Additionally, the Department of Health (DoH) may want to devise quality assurance measures to ensure that home-based care, and other support interventions achieve their intended outcomes. There remains a deficit of evidence on the effect different ART support models have on risky sexual behaviour. Further research is therefore required to determine the role that differentiated treatment support models have on reducing risky behaviour amongst HIV patients.

The UNAIDS 90–90–90 targets and fast-track strategies to end the HIV epidemic by 2030 [36] will undoubtedly require a scale-up of HIV services, specifically HTS and treatment services [1] resulting in more people engaging with the health system, and consequently directly engaging with health personnel providing opportunities to reduce risky sexual behaviour through the employment of BCC strategies. Effective BCC, operationalised through HIV testing and treatment services, is required, focusing on risk reduction counselling and education programmes in an effort to avoid reinfections and reducing the chances of transmission of drug-resistant strains from HIV-infected persons with incomplete viral suppression. Research has indicated that upscaling early initiation onto ART is effective in the prevention of HIV acquisition [37]. A focus of South Africa’s National Strategic Plan is improving the prevention of HIV infections by providing ART at the point of a positive test [1]. This followed the adoption of Universal Test and Treat (UTT) policy in 2016 and could have played a role in the increased uptake of ART between the two surveys, however, what remains a concern are the high rates of risky behaviour exhibited by ART-naïve patients. Counselling services play a crucial role in getting positive patients initiated onto ART but could also amplify the preventative effects of treatment and a suppressed viral load, by positively affecting risky sexual behaviour and contributing to reaching epidemic control by 2030. Improving the effectiveness of BCC, administered through counselling, and delivered through HIV testing and treatment services, is therefore crucial. A review of counselling practices is important if client-health personnel engagement is to be optimised and a reduction of risky sexual activity realised. Encouragingly, previous research has revealed significant reductions in sexual risk behaviour among youth when exposed to HTS in the same region [19] revealing the potential of effectively orchestrated interventions at the community level involving direct contact with health professionals. Given this potential, research should further explore the most effective ways in which BCC strategies in particular could be integrated into HIV testing and treatment services. The quality and type of information provided during health personnel interactions with HIV service users has to be re-evaluated.

The increasing HIV burden facing health workers should be factored into the planned scale-up of testing and treatment services. The analysis of two community-wide surveys has illustrated the increased utilisation of HTS and the subsequent increase in treatment initiation. However, and of concern, the data reveals a reduction in the effectiveness of these services in reducing risky sexual behaviour. Whilst we are unable to determine the true cause of this effect, it remains plausible that an over-burdened health system is finding it difficult to continue to provide comprehensive quality counselling services. The South African National Strategic Plan on HIV, TB and STIs 2017–2021 has identified initiatives aimed at reducing the burden on public health personnel, including the utilisation of self-testing options and treatment adherence clubs [1]. Importantly, opportunities to maximise BCC exposure to clients utilising these services must be sought to ensure that these initiatives not only increase testing and treatment uptake and adherence, but also play a role in promoting responsible sexual behaviour.

This study has limitations, with any analysis regarding sexual behaviour typically conducted with self-reported data affected by recall and social-desirability bias [38]. It is likely that risky sexual behaviour has been underreported, although the impact of this on HIV testing and ART uptake is uncertain. The ART uptake data was self-reported and may also suffer from social-desirability bias. Additionally, cross-sectional data is merely indicative of associations, but not of the causal relationship between HIV interventions and sexual-risk behaviours. This perhaps particularly applies to any relationship between testing and behaviour as there may well be reverse causation whereby more risky patients opt to test more frequently. Behaviour change is linked to multiple, non-linear events and a mapping of causal chains from inputs to impacts for this study was not possible, due to the data only providing insights into the current behaviour trends with regards to sexual risk taking [39]. We did not ask respondents how long they have been on treatment which may have influenced sexual risk behaviour and should be included as a control variable in future studies. Further research should assess the longitudinal causal directionality between risky sexual behaviour, specifically number of partners, and access to HTS. Our research provides mixed results on the correlation between risky sexual behaviour and having accessed HTS, without determining whether an increased number of partners resulted in higher HTS rates, or whether accessing the services itself, had any effect on risky sexual behaviour.

## Conclusions

The adoption of the UNAIDS Fast Track goals places emphasis on the scale up of HIV services with a greater number of people expected to be tested and initiated onto treatment. This presents an opportunity for health personnel to promote responsible sexual behaviour. This study contributes to the debate on the role of HIV testing and treatment services, and health personnel specifically, in influencing sexual behaviour and establishes that current efforts have had a limited positive impact.

## Additional file

**Additional file 1.** Additional tables.

## Data Availability

The datasets generated and/or analysed during the current study are not publicly available due to ongoing state of the study.

## Abbreviations

ART: antiretroviral treatment
BCC: behaviour change communication
EA: enumeration area
HTS: HIV testing services
HIV: human immunodeficiency virus
HTS: HIV testing services
UNAIDS: Joint United Nations Programme on HIV/AIDS
UTT: Universal Test and Treat
