# Supplementary material for: Impact of HIV testing and treatment services on risky sexual behaviour in the uMgungundlovu District, KwaZulu-Natal, South Africa: a cross-sectional study
Source: AIDS Res Ther. 2019 Aug 21;16:20. doi: 10.1186/s12981-019-0237-z (PMC6702730; doi:10.1186/s12981-019-0237-z)
Supplement: Supplementary file 1 — Additional file 1. Additional tables. [file 12981_2019_237_MOESM1_ESM.docx]

**Table S1: Multiple logistic regression models for the association between number of HIV tests and three sexual risk behaviours (adjusted odds ratios with 95% confidence intervals in parentheses) in uMgungundlovu, KwaZulu-Natal, survey period 2014 to 2016.**

|  | **2014/15 Survey** | | | **2015/16 Survey** | | |
| --- | --- | --- | --- | --- | --- | --- |
|  | (19)^  condom use  last partner† | (20)  Transaction  sex‡ | (21)  Sex partners  12 mo. § | (22)  condom use  last partner† | (23)  Transaction  sex‡ | (24)  Sexual partners  12 mo. § |
| 1 HIV test previous 12 mo. (vs. > 5 tests) | 1.08  (0.82-1.43) | 1.71***  (1.27-2.30) | 0.66*  (0.46-0.95) | 1.05  (0.86-1.27) | 0.88  (0.64-1.20) | 0.95  (0.76-1.19) |
| 2 HIV tests previous 12 mo. (vs. > 5 tests) | 1.20  (0.92-1.55) | 1.30  (0.91-1.85) | 0.68*  (0.48-0.96) | 1.36**  (1.11-1.66) | 1.75***  (1.27-2.40) | 1.19  (0.94-1.49) |
| 3 HIV tests previous 12 mo. (vs. > 5 tests) | 1.12  (0.85-1.48) | 1.76***  (1.19-2.60) | 0.75  (0.51-1.11) | 1.16  (0.96-1.41) | 1.76***  (1.33-2.33) | 0.93  (0.72-1.19) |
| 4 HIV tests previous 12 mo. (vs. > 5 tests) | 0.82  (0.60-1.13) | 1.43  (0.96-2.11) | 0.91  (0.58-1.42) | 1.26*  (1.01-1.58) | 1.24  (0.88-1.75) | 1.02  (0.78-1.33) |
| 15-24 yrs. (vs. 35-49 yrs.) | 0.78*  (0.63-0.97) | 0.82  (0.62-1.09) | 1.95***  (1.38-2.74) | 1.05  (0.86-1.27) | 0.97  (0.77-1.21) | 1.78***  (1.41-2.25) |
| 25-34 yrs. (vs. 35-49 yrs.) | 1.16  (0.94-1.43) | 1.05  (0.85-1.32) | 1.88***  (1.35-2.62) | 1.35**  (1.13-1.62) | 1.15  (0.93-1.42) | 1.58***  (1.26-1.99) |
| Male (vs. female) | 0.77**  (0.65-0.91) | 0.83  (0.66-1.03) | 6.85***  (5.31-8.85) | 0.74***  (0.65-0.84) | 0.96  (0.82-1.14) | 4.45***  (3.77-5.24) |
| Married (vs. unmarried) | 1.25  (0.89-1.77) | 0.62*  (0.44-0.87) | 0.17***  (0.09-0.34) | 1.10  (0.85-1.41) | 0.65*  (0.49-0.88) | 0.31***  (0.21-0.45) |
| Incomplete secondary school (vs. tertiary) | 1.31  (0.93-1.85) | 2.21***  (1.29-3.76) | 0.68  (0.42-1.12) | 1.25  (0.98-1.60) | 0.98  (0.69-1.40) | 1.2  (0.88-1.64) |
| Complete secondary school (vs. tertiary) | 1.22  (0.88-1.68) | 1.72*  (1.03-2.85) | 0.53***  (0.35-0.82) | 1.11  (0.87-1.41) | 1.07  (0.78-1.47) | 1.15  (0.85-1.55) |
| away > 1 mo. in previous year (vs. < 1mo. previous year) | 1.24  (0.90-1.70) | 1.21  (0.86-1.70) | 1.01  (0.67-1.52) | 0.90  (0.70-1.17) | 0.9  (0.64-1.27) | 1.59***  (1.24-2.04) |
| Highly food insecure (vs. food secure) | 0.88  (0.69-1.11) | 0.56***  (0.40-0.79) | 1.21  (0.91-1.61) | 0.88*  (0.75-1.03) | 0.53***  (0.37-0.74) | 1.28*  (1.03-1.60) |
| Moderate food insecure (vs. food secure) | 1.05  (0.78-1.41) | 0.75  (0.50-1.12) | 1.3  (0.85-1.99) | 0.76**  (0.63-0.91) | 0.47***  (0.32-0.69) | 1.43***  (1.12-1.83) |
| Partner HIV positive (vs. don’t know partner status) | 0.63*  (0.43-0.92) | . | . | 1.06  (0.77-1.45) | . | . |
| Partner not HIV positive (vs. don’t know partner status) | 1.16  (0.90-1.51) | . | . | 1.22  (0.93-1.60) | . | . |
| Duration of last relationship (continuous) | 1.03**  (1.01-1.05) | . | . | 1.09***  (1.07-1.10) | . | . |
| Pseudo R^2^ | 0.04 | 0.03 | 0.21 | 0.07 | 0.04 | 0.16 |
| n | 6316 | 6553 | 5151 | 8142 | 8167 | 7204 |

Notes: ^The numbers in the brackets refer to the Multivariate regression model number. *** p <0.001, ** p <0.01, * p <0.05. † Condom use with most recent sexual partner in the last 12 months (0 = consistent condom use/always used condoms; 1 = inconsistent condom use/sometimes or never used condoms). ‡ engaging in transactional sex (0 = not giving or receiving goods or money for sex in the previous year; 1 = have given or received goods or money for sex in the previous year). § number of sexual partners in the previous year (0 = 0 to 1 sexual partner in the previous year; 1 = 2 or more sexual partners in the previous year).

**Table S2: Multiple logistic regression models for the association between types of HIV treatment support and three sexual risk behaviours (adjusted odds ratios with 95% confidence intervals in parentheses) in uMgungundlovu, KwaZulu-Natal, survey period 2014 to 2016.**

|  | **2014/15 survey** | | | **2015/16 survey** | | |
| --- | --- | --- | --- | --- | --- | --- |
|  | (25) ^  condom use  last partner† | (26)  Transaction  sex‡ | (27)  Sex partners  12 mo. § | (28)  condom use  last partner† | (29)  Transaction  sex‡ | (30)  Sexual partners  12 mo. § |
| Received emotional support (vs. No emotional support) | 0.79  (0.60-1.03) | 0.68  (0.43-1.07) | 1.57  (0.88-2.80) | 0.95  (0.72-1.24) | 0.38***  (0.23-0.65) | 0.65*  (0.43-0.97) |
| Had a treatment buddy (vs. did not have a treatment buddy) | 0.70  (0.49-1.01) | 0.60*  (0.38-0.96) | 1.09  (0.53-2.22) | 0.73  (0.53-1.01) | 0.31***  (0.14-0.65) | 0.46*  (0.26-0.79) |
| Received home-based support (vs. did not receive home-based support) | 1.63  (0.75-3.55) | 1.24  (0.65-2.37) | 0.55  (0.19-1.60) | 2.24***  (1.56-3.23) | 5.40***  (3.11-9.39) | 1.97*  (1.16-3.36) |
| 15-24 yrs. (vs. 35-49 yrs.) | 0.86  (0.52-1.42) | 0.96  (0.61-1.49) | 1.25  (0.56-2.81) | 1.50  (0.98-2.31) | 1.34  (0.83-2.16) | 1.61  (0.95-2.73) |
| 25-34 yrs. (vs. 35-49 yrs.) | 1.10  (0.84-1.44) | 0.86  (0.62-1.19) | 1.93*  (1.09-3.41) | 1.40**  (1.11-1.77) | 1.27  (0.97-1.66) | 1.27  (0.91-1.78) |
| Male (vs. female) | 0.81  (0.59-1.12) | 0.96  (0.67-1.39) | 4.38***  (2.81-6.82) | 0.79  (0.59-1.04) | 1.12  (0.81-1.54) | 3.78***  (2.67-5.35) |
| Married (vs. unmarried) | 0.64*  (0.42-0.97) | 0.48*  (0.27-0.86) | 0.22***  (0.08-0.60) | 0.82  (0.59-1.14) | 0.81  (0.52-1.26) | 0.44*  (0.23-0.86) |
| Incomplete secondary school (vs. tertiary) | 1.70  (0.82-3.51) | 1.77  (0.74-4.27) | 1.06  (0.36-3.11) | 1.75  (0.98-3.14) | 0.63  (0.25-1.56) | 1.38  (0.50-3.81) |
| Complete secondary school (vs. tertiary) | 1.52  (0.72-3.21) | 1.3  (0.53-3.19) | 0.68  (0.23-2.07) | 1.37  (0.74-2.54) | 0.63  (0.25-1.57) | 1.23  (0.45-3.36) |
| away > 1 mo. in previous year (vs. < 1mo. previous year) | 1.61  (0.97-2.67) | 1.31  (0.81-2.12) | 0.53  (0.23-1.23) | 0.70  (0.44-1.12) | 1.01  (0.49-2.10) | 1.93*  (1.09-3.42) |
| Highly food insecure (vs. food secure) | 0.88  (0.54-1.44) | 0.62*  (0.40-0.96) | 1.59  (0.98-2.60) | 0.81  (0.62-1.05) | 0.57***  (0.39-0.82) | 1.67*  (1.11-2.51) |
| Moderate food insecure (vs. food secure) | 0.88  (0.54-1.44) | 0.98  (0.56-1.72) | 1.15  (0.40-3.29) | 0.77  (0.56-1.06) | 0.60*  (0.37-0.98) | 1.62  (0.98-2.66) |
| Partner HIV positive (vs. don’t know partner status) | 0.77  (0.50-1.20) | . | . | 1.12  (0.73-1.71) | . | . |
| Partner not HIV positive (vs. don’t know partner status) | 0.82  (0.58-1.17) | . | . | 0.84  (0.56-1.26) | . | . |
| Duration of last relationship (continuous) | 1.01  (0.98-1.03) | . | . | 1.06***  (1.04-1.09) | . | . |
| Pseudo R^2^ | 0.03 | 0.04 | 0.15 | 0.07 | 0.16 | 0.14 |
| n | 2243 | 2319 | 1722 | 2701 | 2712 | 2320 |

Notes: ^The numbers in the brackets refer to the Multivariate regression model number. *** p <0.001, ** p <0.01, * p <0.05. † Condom use with most recent sexual partner in the last 12 months (0 = consistent condom use/always used condoms; 1 = inconsistent condom use/sometimes or never used condoms). ‡ engaging in transactional sex (0 = not giving or receiving goods or money for sex in the previous year; 1 = have given or received goods or money for sex in the previous year). § number of sexual partners in the previous year (0 = 0 to 1 sexual partner in the previous year; 1 = 2 or more sexual partners in the previous year).
